# Development of Predictive Model of Surgical Case Durations Using Machine Learning Approach

**DOI:** 10.1007/s10916-025-02141-y

**Published:** 2025-01-14

**Authors:** Jung-Bin Park, Gyun-Ho Roh, Kwangsoo Kim, Hee-Soo Kim

**Affiliations:** 1 Department of Anesthesiology and Pain Medicine, Seoul National University Hospital, Seoul National University College of Medicine, Seoul, Republic of Korea; 2 Interdisciplinary Program of Medical Informatics, Seoul National University, Seoul, Republic of Korea; 3 Department of Transdisciplinary Medicine, Institute of Convergence Medicine With Innovative Technology, Seoul National University Hospital, Seoul, Republic of Korea; 4 Department of Medicine, College of Medicine, Seoul National University, Seoul, Republic of Korea

**Keywords:** Operating rooms, Machine learning, Hospital departments, Surgical procedures, Clinical decision-making

## Abstract

Optimizing operating room (OR) utilization is critical for enhancing hospital management and operational efficiency. Accurate surgical case duration predictions are essential for achieving this optimization. Our study aimed to refine the accuracy of these predictions beyond traditional estimation methods by developing Random Forest models tailored to specific surgical departments. Utilizing a comprehensive dataset, we applied several machine learning algorithms, including RandomForest, XGBoost, Linear Regression, LightGBM, and CatBoost, and assessed their performance using Mean Absolute Error (MAE), Root Mean Squared Error (RMSE), and R-Squared (R^2^) metrics. Our findings highlighted that Random Forest models excelled in department-specific applications, achieving an MAE of 16.32, an RMSE of 31.19, and an R^2^ of 0.92, significantly outperforming general models and conventional estimates. This improvement emphasizes the advantage of customizing models to fit the distinct characteristics and data patterns of each department. Additionally, our SHAP-based feature importance analysis identified morning operation timing, ICU ward assignments, operation codes, and surgeon IDs as key factors influencing surgical duration. This suggests that a detailed and nuanced approach to model development can substantially increase prediction accuracy. By providing a more accurate, reliable tool for predicting surgical case durations, our department-specific Random Forest models promise to enhance surgical scheduling, leading to more effective OR management. This approach underscores the importance of leveraging tailored, data-driven models to improve healthcare outcomes and operational efficiency.

**Supplementary Information:**

The online version contains supplementary material available at 10.1007/s10916-025-02141-y.

## Introduction

Optimizing operating room utilization is a pivotal aspect in hospital management strategy. It is fundamental to strategically arrange surgery schedules taking into account available hospital beds and personnel, placing patient safety as the top priority. The primary and crucial step in arranging operating room is to assess and predict surgical case duration [1–4]. However, predicting surgery duration is difficult due to numerous uncertain factors such as patient’s condition, change of surgical plan, surgical instrument issues, and unexpected bleeding [5,6]. In many hospitals, operating rooms are managed based on the common approaches of predicting surgical duration by applying average durations for specific procedures or based on the anesthesiologist’s subjective outlook [7,8]. Inaccurate estimates of surgical case duration result in increased hospital operating expenses, extended working hours for healthcare staff, leading to dissatisfaction and fatigue and raised the risk of medical errors [9–12]. Furthermore, it results in longer patient wait times, fasting times, and heightened dissatisfaction among patients and their families. This emphasizes the importance of development of more accurate and reliable method for predicting surgical duration.

Application of a machine learning algorithm in operating room management has been suggested to enable significant improvement in case duration prediction by using various input variables or feature [13–17]. Bartek et al. reported the machine learning based model for predicting surgical duration enhanced the prediction accuracy to 39% [18]. Strömblad et al. showed that the implementation of a predictive model improved accuracy in predicting surgical procedures and reduced patient wait time in a presurgical area [14]. However, there are only few studies reporting models tailored for each specific surgical department. Also, in the case of a teaching hospital, the fact that the same surgical procedures are performed by new team members with new residents each month, rather than a fixed team, has often not been considered in most studies [3]. This overlooks the potential impact of scheduled surgery days, weekly variations, and monthly changes on surgical duration.

In this study, we aimed to develop both general and department-specific surgical duration prediction models using a machine learning approach. We hypothesized that improving model performance could be achieved by taking into account the characteristics of each department, as well as the timing and seasonal factors of scheduled surgeries in comparison to the surgeon’s expectations.

## Methods

### Ethics Statements

This retrospective study was approved by the Institutional Review Board (IRB) of Seoul National University Hospital, Seoul, Korea, (Number 2306–164–1443; Date of approval, 29 June 2023).

### Data Sources

**Fig. 1:**
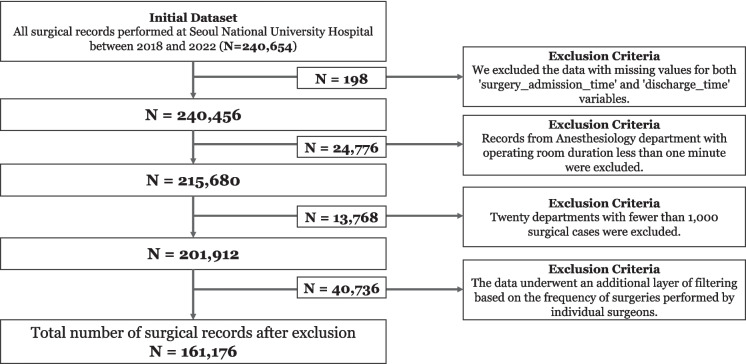
Flowchart for data Filtering process

This study utilizes a comprehensive dataset from Seoul National University Hospital, initially comprising 240,654 surgical records from 2018 to 2022. The dataset underwent several sequential refinement steps as outlined in Fig. 1. First, 198 records with missing values for critical timestamps ('surgery_admission_time' and 'discharge_time' variables) were excluded. Second, 24,776 records non-operating room schedules, which operate independently from the operating room schedule but were registered in the surgical schedule to fulfill administrative requirements. Third, twenty departments with fewer than 1,000 surgical cases were excluded (13,768 cases), including Musculoskeletal Rehabilitation(729 cases), Radiology(890 cases), Pediatric Cardiology(710 cases), and various other subspecialties such as Pediatric Hematological Oncology and Nephrology. This filtering ensured adequate sample sizes for meaningful analysis.

The remaining data were organized into 17 key departments, as detailed in Table 1. This organization involved consolidating related surgical services: General Surgery incorporated procedures from eight divisions (including General Surgery, Colon and Rectal Surgery, Gastrointestinal Surgery, Hepatobiliary Pancreatic Surgery, and Breast Endocrine Surgery), Orthopedics combined three related units, and Plastic Surgery merged with Transplant Vascular Surgery. The table presents the number of cases, mean duration, and standard deviation of duration for each consolidated surgical department. The final refinement step involved analyzing surgical frequency by individual surgeons, excluding cases that fell below the 85th percentile threshold of surgical volume (40,736 cases), ensuring the dataset represented the most active surgical practices. These systematic refinements resulted in a final dataset of 161,176 surgical records, categorized into 131,489 adult and 29,687 pediatric surgeries.

**Table 1:** Adult and Pediatric Departments with Case Statistics

Type	Department	Cases	Mean Duration (min)	Std duration (min)
Adult	General Surgery	36,424	155.72	105.05
Adult	Cardiovascular Thoracic Surgery	9,341	233.43	143.51
Adult	Neurosurgery	7,187	239.46	126.33
Adult	Orthopedics	16,617	133.93	89.45
Adult	Otolaryngology	11,772	139.02	110.18
Adult	Ophthalmology	17,437	57.83	34.14
Adult	Urology	12,741	115.44	76.87
Adult	Obstetrics & Gynecology	11,510	131.53	84.12
Adult	Plastic Surgery	8,460	139.34	123.08
Pediatric	Pediatric Surgery	4,021	97.25	83.72
Pediatric	Pediatric Thoracic Surgery	1,876	269.96	180.04
Pediatric	Pediatric Neurosurgery	1,166	296.25	149.77
Pediatric	Pediatric Orthopedics	3,905	143.96	94.02
Pediatric	Pediatric Otolaryngology	5,763	84.34	68.92
Pediatric	Pediatric Ophthalmology	7,857	56.66	20.23
Pediatric	Pediatric Urology	2,975	114.53	90.38
Pediatric	Pediatric Plastic Surgery	2,124	115.22	79.54

### Preprocessing

In our study, Table 2 outlines the dataset features, with detailed values, ranges, and statistics provided in Supplementary Table S1. We customized our preprocessing approach for each variable with missing data to ensure dataset completeness and integrity. For numerical variable 'BMI' with 4.83% missing values, we used the KNN Imputer method with n_neighbors = 5 for imputation, leveraging the KNN algorithm to identify nearest neighbors and impute missing values based on their mean. This method is particularly effective for nonlinear data, leveraging patterns within the dataset to accurately fill in missing values [19]. Prior to model development, we conducted a correlation analysis of both numerical and categorical variables. This analysis revealed high similarity between admission department and surgical department (83.88%). To address this collinearity issue, we chose to retain surgical department and exclude admission department from our model, as surgical department is more directly related to the actual surgical procedure.

**Table 2:** Surgery and Patient Data Features

Feature	Type	Description
Gender	Categorical	Patient's gender (M, F)
Age	Numeric	Patient's age
BMI	Numeric	Patient's Body Mass Index
Asa class	Categorical	ASA(American Society of Anesthesiologists) classification before surgery
Condition source value	Categorical	Patient's diagnosis
Surgeon ID	Categorical	Unique ID of the surgeon who performed the surgery
Surgical department	Categorical	Department where surgery was conducted
Previous surgery	Categorical	Whether the patient had the same surgery before (Yes or No)
Day of the week	Categorical	Day of the week when surgery was performed
Week of the month	Categorical	Week of the month when surgery was performed
Month	Categorical	Month when surgery was performed
Operation code	Categorical	Code for the surgical procedure
Anesthesia type	Categorical	Type of anesthesia used
Emergency status	Categorical	Whether the surgery was an emergency (Yes or No)
Operation timing	Categorical	Order of the surgery
Surgery room	Categorical	Number of the surgery room
Division	Categorical	Admission/Outpatient
Ward	Categorical	Ward where the patient was admitted

Categorical variables were encoded based on their characteristics and the number of unique values within each variable. One-hot encoding was used for categorical variables with a lower number of distinct categories, such as temporal variables (month: 12 categories; day of the week: 7 categories; week of the month: 5 categories), patient and procedure characteristics (gender: 2 categories; division: 3 categories; anesthesia type: 8 categories; previous surgery: 2 categories; emergency status: 2 categories; operation timing: 14 categories), and facility-related variables (surgical department: 17 categories; ward: 71 categories; surgery room: 53 categories). This encoding method preserves the categorical nature of the variables without imposing excessive computational burden.

For categorical variables with a high number of unique values, such as operation code (3,061 unique values), final operation name (18,993 unique values), and condition source value (6,175 unique values), we employed binary encoding to efficiently represent the categories while managing dimensionality. Binary encoding represents each unique category using log2(n) binary digits, where n is the number of unique values. This approach significantly reduces the feature space compared to one-hot encoding, which would have created an excessive number of new columns.

### Model Development

Our study developed predictive models using multiple machine learning algorithms for regression tasks. The analysis was conducted using Python 3.8 with the following packages: scikit-learn (version 1.0.2) for RandomForest [20], and Linear Regression [21] implementations, XGBoost [22] (version 1.5.0), LightGBM [23] (version 3.3.2), and CatBoost [24] (version 1.0.5). The dataset was split into training (80%) and testing (20%) sets using stratified sampling based on the 'surgical department' attribute to maintain proportional representation across departments. We employed fivefold cross-validation on the training set, where in each iteration, 20% of the training data served as the validation set and the remaining 80% for training. Performance metrics were averaged across the 5 iterations to assess model generalization.

**Fig. 2:**
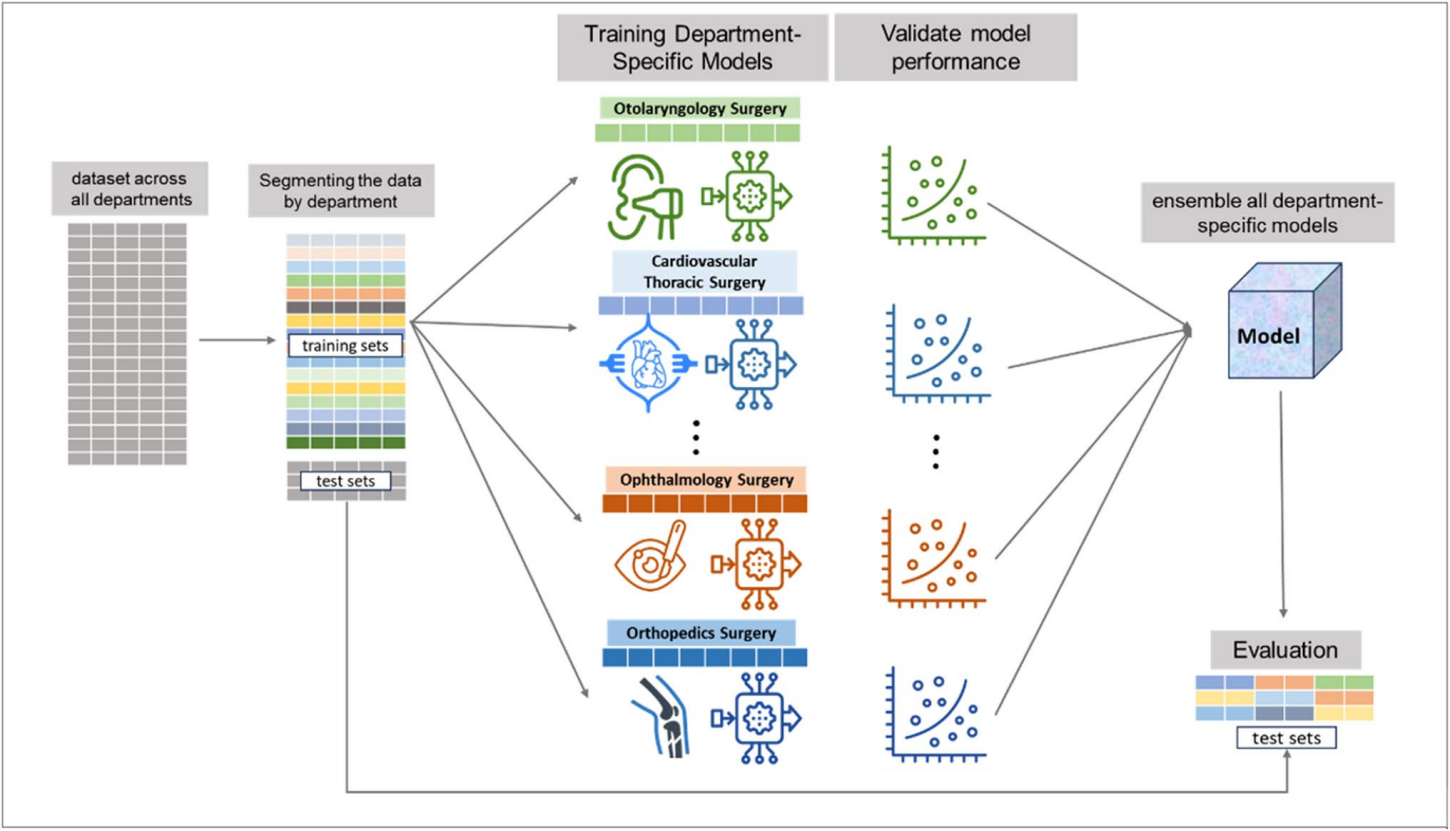
Department-Specific Modeling Approach

We utilized all available features without feature selection, allowing the models to determine feature importance during training. Hyperparameter tuning was performed using grid search across multiple parameters for each algorithm. For RandomForest, we tuned n_estimators ranging from 100 to 300, max_depth of 10, 20, and None, and min_samples_split of 2, 5, and 10. In XGBoost, we optimized learning rates of 0.01 and 0.1, max_depth of 3, 6, and 9, and n_estimators of 100 and 200. The LightGBM parameters included num_leaves of 31 and 63, learning rates of 0.01 and 0.1, and n_estimators of 100 and 200. For CatBoost, we tuned iterations between 100 and 200, learning rates of 0.01 and 0.1, and depth values of 6 and 8. Our approach segmented the dataset based on the 'surgical department' attribute, enabling department-specific model training. For each department, we evaluated the algorithms using Mean Absolute Error (MAE), Root Mean Squared Error (RMSE), and R-Squared (R2) metrics. The best-performing model for each department was selected based on these metrics. The final prediction system utilized department-specific models where each department's model was responsible for predictions within its respective surgical department in the test dataset(Fig. 2).

To analyze feature importance, we employed SHAP (SHapley Additive exPlanations) values using the TreeSHAP algorithm, which is specifically optimized for tree-based models. The shap Python package (version 0.41.0) was used to calculate these values. SHAP values provide a theoretically grounded approach to feature importance by computing the marginal contribution of each feature across all possible feature combinations. This method offers both global and local interpretability, considering feature interactions while ensuring fair attribution of feature contributions based on game theory principles.

## Results

### Comprehensive Evaluation of Machine Learning Algorithms

In our study comparing methods for predicting surgical durations, we evaluated two different modeling approaches: (1) general models trained on the entire dataset across all departments, and (2) department-specific models where separate models were trained for each surgical department. All performance metrics were calculated using the test set (20% of the data) that was completely separate from the training process. For general models using the combined data from all departments, Random Forest achieved an MAE of 34.05, RMSE of 57.45, and an R2 of 0.72. The department-specific approach, where individual Random Forest models were trained for each surgical department, showed even better performance with an MAE of 16.32, RMSE of 31.19, and an R2 of 0.92. All other algorithms showed consistently better performance when trained on department-specific data compared to their general model Table 3.

**Table 3:** Performance Comparison between General Models and Department-Specific Models on Test Set

Algorithm	General Models			Department-Specific Models		
	**MAE**	**RMSE**	**R2**	**MAE**	**RMSE**	**R2**
**Random Forest**	34.05	57.45	0.72	**16.32**	**31.19**	**0.92**
**XGBoost**	36.20	59.31	0.70	27.04	43.11	0.84
**Linear Regression**	60.21	87.48	0.36	50.22	76.98	0.50
**LightGBM**	38.37	61.61	0.68	28.91	46.73	0.82
**Catboost**	37.65	60.75	0.69	29.59	46.87	0.81

### Analysis of Feature Importance

**Fig. 3:**
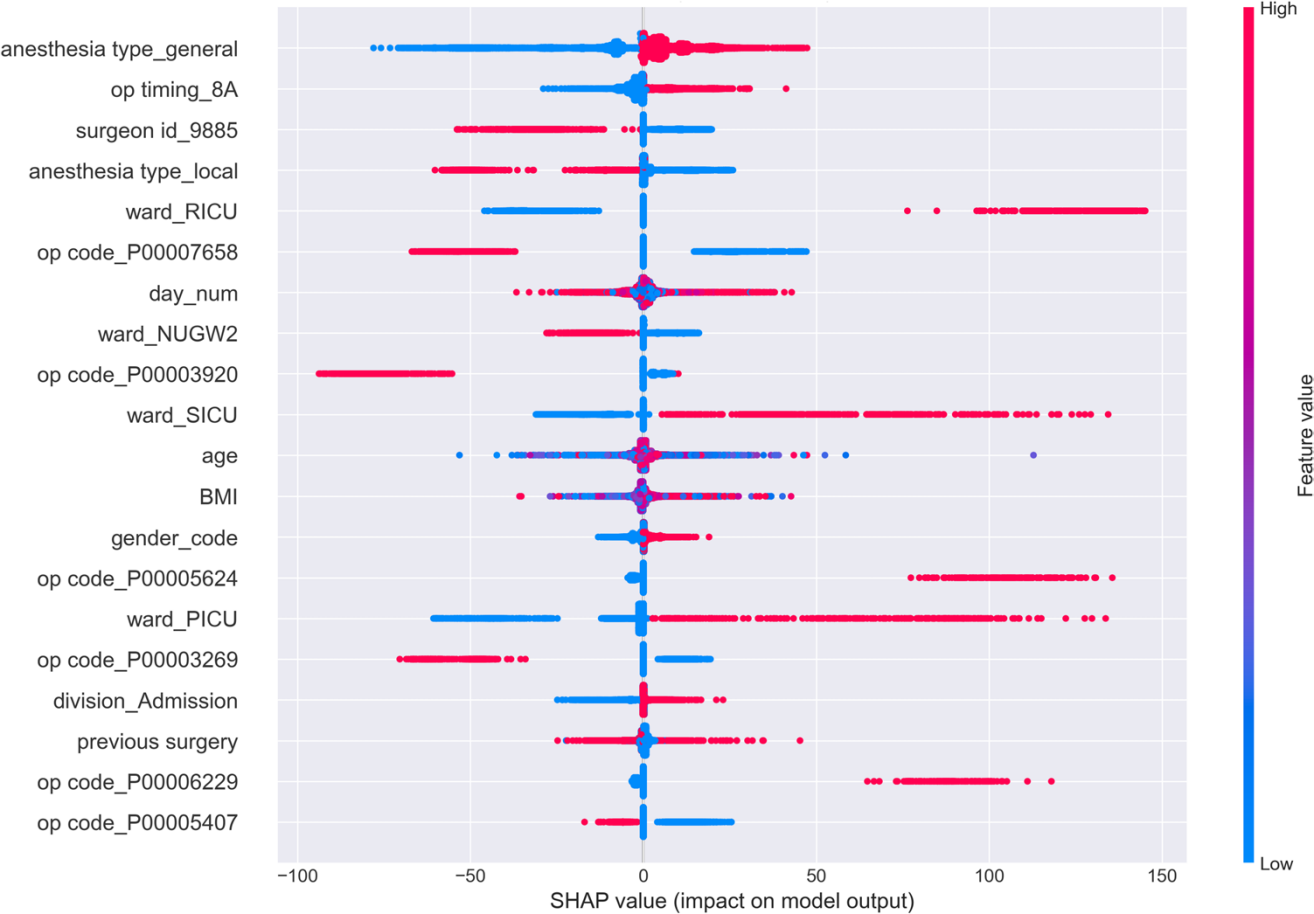
Feature importance Across All Departments(Top 20 Features)

In our research, we analyzed feature importance using SHAP values to understand how different features influence surgical duration predictions (Fig. 3). The analysis revealed morning surgeries (operation timing 8A) as the most influential predictor of surgical duration. The type of ward, specifically ICU assignments, emerged as the next most important category of features, suggesting that the level of care required by patients significantly affects surgical times. Specific operation codes and surgeon IDs also showed high influence on surgical duration predictions, indicating the significance of procedure types and individual surgeon characteristics. Patient demographics including age and BMI also demonstrated substantial importance in predicting surgical duration.

### Comparison of Machine Learning Models and Surgeon Estimates

In our study, we evaluated three prediction methods for surgical duration: department-specific models, general models, and Surgeon's Estimates. The department-specific models proved most accurate with an MAE of 16.67, RMSE of 31.66, and R^2^ of 0.92(Table 4). General models showed less precision (MAE of 34.04, RMSE of 57.45, R^2^ of 0.72) due to their broad application across departments. Surgeon's Estimates were least accurate, with an MAE of 70.81, RMSE of 94.34, and R^2^ of 0.43, highlighting the limitations of traditional estimations.

**Table 4:** Performance Metrics of Surgical Duration Prediction Models

	**MAE**	**RMSE**	**R2**
Department-Specific Model	**16.32**	**31.19**	**0.92**
General Model	34.05	57.45	0.72
Surgeon's Estimate	70.81	94.34	0.43

**Fig. 4:**
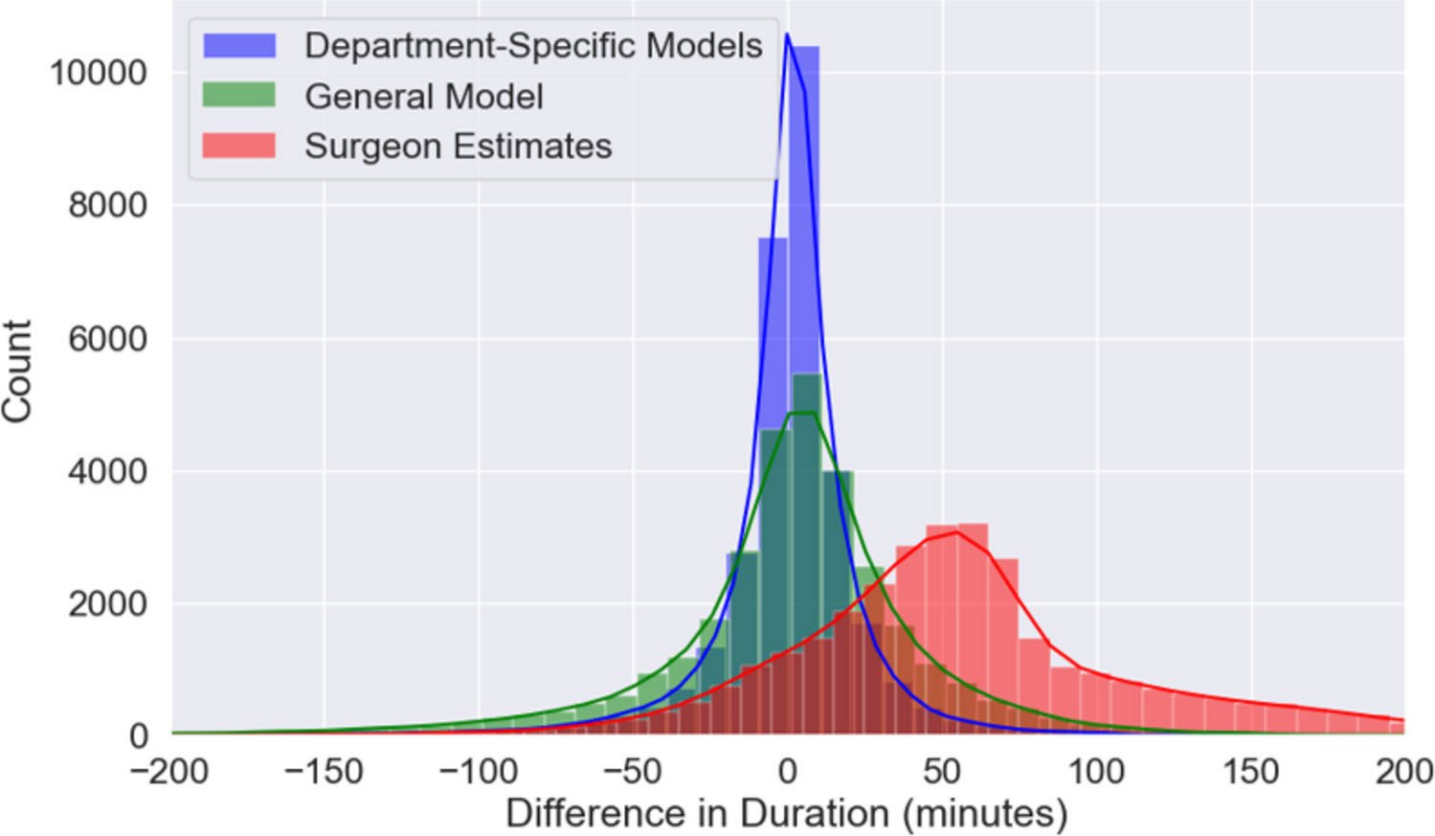
Distribution of Differences Between Predicted and Actual Surgical Durations

A histogram visually represented these differences, showing the tightest distribution for department-specific models, indicating higher accuracy compared to the wider distributions of general models and Surgeon's Estimates(Fig. 4).

### Differences in Prediction Performance by Department

**Fig. 5:**
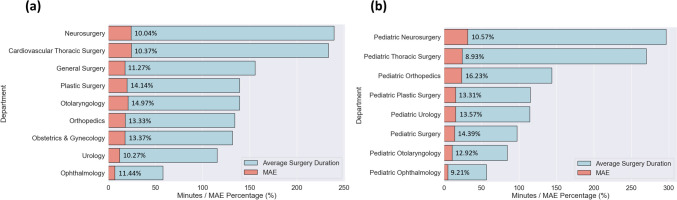
Variations in Average Surgery Duration, MAE, and MAPE by Department, (a) Adult Departments; (b) Pediatric Departments

In our research, we conducted a detailed evaluation of the accuracy of surgical duration prediction across various specialties, including both adult and pediatric cases. We analyzed differences in Mean Absolute Percentage Error (MAPE) across different medical departments (Fig. 5).

Among the entire range of specialties, we noted an average Mean Absolute Percentage Error (MAPE) of approximately 12.26%. Adult Neurosurgery has an MAE of 24.03 min with a MAPE of 10.04%, while Cardiovascular Thoracic Surgery follows at 23.35 min and 10.37% MAPE. Pediatric Plastic Surgery, Urology, General Surgery, Otolaryngology, and Ophthalmology have durations from 115.20 to 56.63 min and MAPEs between 13.31% and 9.21%.

### Weekly and Monthly Trends in Surgical Predictions

**Fig. 6:**
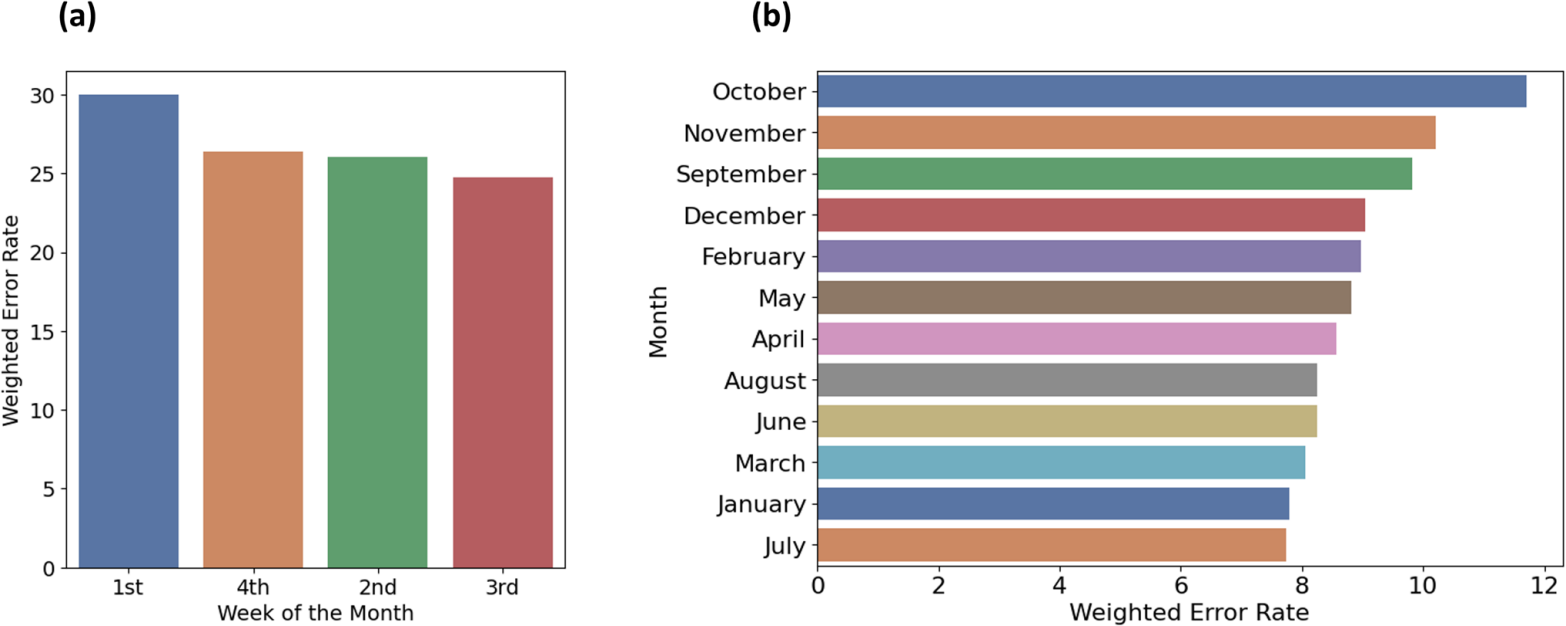
Weighted Error Rate by surgery date, (a) Weighted Error Rate by Week of the Month; (b) Weighted Error Rate by Month

We methodically assessed the accuracy of surgical duration predictions, focusing on weekly and monthly variations(Fig. 6). Our department-specific predictive models were used, with analysis concentrated on the top 10% of data points with the highest prediction errors. To address data imbalance, we conducted a weighted error rate analysis, adjusting error rates based on sample sizes.

$$Weighted Error Rate= \frac{Sample Size \times Mean Absolute Error}{Sample Size of the Category}$$

Results showed higher error rates in the first week of each month (30.02%) and in October (11.72%). Lower error rates were observed in subsequent weeks and in months like July and January. Overall, our analysis highlights the need for improved scrutiny in surgical scheduling, particularly at the beginning of each month.

## Discussion

We developed and evaluated ensemble learning-based case duration prediction models that demonstrated higher accuracy compared to surgeon’s estimates. Among the developed models, notable improvements in predictive accuracy were observed in the department-specific Random Forest models, tailored to the unique characteristics of each department, with an MAE of 16.32 min.

Previous studies have reported that the average cost for operating room charges was $62 per minute, excluding fees for surgeons and anesthesia providers [18]. If precise prediction of surgical duration enables efficient utilization of operating rooms, it would result a substantial reduction in costs [25,26]. These cost savings would include staffing overtime pay, operating room overall fees, unnecessary hospitalizations due to delays in outpatient surgery, costs associated with extended length of stay, and the cost of unfilled operating rooms and institutional profit [4,27,28]. For example, delays caused by insufficient preoperative preparation may leave the operating room unoccupied for 30 min, during which a procedure such as neonatal open inguinal hernia repair could have been performed, with a total cost of approximately $2,000 ($66 per minute). Similarly, surgeries exceeding scheduled hours result in unnecessary patient hospitalization cost of $200 per day and overtime pay of at least $650 per hour ($22 per minute) for operating room staff. In addition, preoperative extended fasting time is considered one of the major contributors to the development of postoperative cognitive dysfunction, postoperative delirium, postoperative gastrointestinal discomfort, insulin resistance, postoperative nausea and vomiting, and patient dissatisfaction [29–32]. Accurate prediction of surgical duration and personalized adjustment of preoperative fasting time for patients could have positive impact on patient safety and postoperative outcome through the reduction of preoperative fasting duration [31].

Our study highlighted the superiority of department-specific Random Forest models in predicting surgical durations across different hospital specialties, achieving an MAE of 16.32 min, an RMSE of 31.19 min, and an R^2^ of 0.92, markedly outperforming the Random Forest model from previous research which had an R^2^ of 85% and an MAE of 30.2 minutes [33]. Our results, with a significant improvement indicated by a lower MAPE of approximately 12.26%, underscore the advantages of customized models over generic ones in accurately forecasting surgical times [25,34]. This improvement showcases the potential of specialized models in capturing the unique aspects of surgical operations, patient demographics, and operational dynamics that generalized models may miss. Furthermore, the Random Forest's proficiency in managing complex data interactions and preventing overfitting provides a critical edge in the variable-rich environment of surgical departments, affirming the value of bespoke, department-oriented modeling approaches in enhancing surgical duration prediction accuracy.

In this study, feature importance identified that operation code and ward were significantly influencing the prediction of surgical duration. This finding is interpretable and compatible with previous research that operation type were important for predicting surgical duration [14,15,17,25]. In our institution, we operate separate wards for short-term and outpatient admissions as well as for long-term hospital stays. These factors appeared to have influenced predictability. Other interesting features with an important impact included the temporal aspect of surgery such as month, day of the week, week of the month, op timing in our study. These could be associated with variations in hospital workflows, staff fatigue, and resource availability throughout the day and week. The increased error rates during the first week of each month coincide with the period of resident rotation. During this time, new residents may not yet be familiar with the workflow, leading to delays in surgery end times or extended durations between surgery end and anesthesia start times. Furthermore, the notable decline in surgical efficiency towards the year's end, as reflected in the increased error rates in October and November, can be attributed to the reduced workforce due to the preparation for the board certifical exam by the fourth-year residents. These transitions likely disrupt the regular flow and efficiency of surgical operations. Our findings advocate for a more dynamic approach to surgical scheduling, particularly during periods of staff transitions in teaching hospitals.

We observed variations in prediction accuracy depending on the department and whether the patients were pediatric or adult. Procedures that are well-defined, repetitive or conducted as part of examinations, and operated by a fixed team, such as pediatric thoracic surgery or ophthalmology, exhibited considerably low MAPE. However, surgeries in Pediatric Orthopedics, otolaryngology or plastic surgery, which may necessitate changes in surgical plans based on frozen biopsy results, require reconstruction surgery depending on the size of the defect, or need for reoperation after flap failure, exhibited lower predictive accuracy. In the case of pediatric orthopedics in our institution, where uncooperative children with rare and complex musculoskeletal diseases are treated, surgical plans often undergo changes following a comprehensive examination under general anesthesia, evolving into unexpectedly complex surgeries, contributing to the lower predictive accuracy. Overall, the challenge in predicting surgical durations in the pediatric patient care department arose from several factors at our institution. Many patients diagnosed with rare diseases contributed to surgical complexity. Additionally, frequent alterations in surgical plans occurred after comprehensive evaluations under general anesthesia in uncooperative children. The difficulty in surgical preparation included challenges such as airway management, line insertion, and adopting surgical positions for patients with restrictive range of motion.

We compared the performance of developed models to surgeon estimates, which holds significance as, the scheduling of surgical cases at out institution is based on estimates provided by primary surgeons. Their predictions tend to provide conservative estimate to anesthesiologists, taking into account variables such as the patient’s underlying disease and specific surgical circumstances [35]. Furthermore, even though they could input surgical times in one-minute intervals, the practice of selecting values at 30-min intervals might be contributed to errors, resulting in an average discrepancy of approximately 70 min from the actual surgical duration ^36^. These inaccurate estimates could led to inefficiencies in operating room management, resulting in several surgeries being delayed during off-hours when there was insufficient staffing, and surgeries being delayed into the nighttime.

This study had several limitations. First, this study was a single-center retrospective study to generalize the results. There would be limitations in applying the models to other hospitals due to differences in the operating room management system. Second, intraoperative data, specific surgical details, and detailed underlying diseases of each patient were not included in input variables. However, our model, developed with adequate predictive capabilities despite limited retrospective data, suggested that a machine learning-based model for operating room scheduling could be further refined with using detailed medical records. Additionally, this model could be incorporated as a standard practice in healthcare settings after validation. Thirdly, this study excluded the bottom 15% of cases with low surgical counts, primarily performed by fellows or clinical assistants who are temporary trainees not assigned to regular surgical schedules or operating rooms per institutional policy. These cases, accounting for less than 15% of the dataset, were excluded to minimize their influence as outliers and to enhance the model’s overall performance. While this exclusion may limit the generalizability of the model to non-standard surgical cases, the primary focus of this study was to optimize predictions for standard operating schedules and improve hospital efficiency. Further studies are needed to enhance the model’s generalizability and achieve more balanced utility in real-world surgical scheduling scenarios. Lastly, our models were not externally validated and the potential benefits of surgical duration prediction using machine learning techniques were not evaluated.

In conclusion, we developed machine learning-based model for predicting surgical case durations outperformed surgeon estimates, especially when department-specific. The results from our models provide a valuable tool for enhancing operational efficiency. By accurately predicting surgical times, we anticipate that hospitals can optimize the utilization of operating rooms, improve staffing and resource management, and ultimately improve patient care and satisfaction. The methodologies and insights derived from this research can also be adapted and applied to other aspects of healthcare management, paving the way for more data-driven, efficient, and patient-centric healthcare systems.

## Supplementary Information

Below is the link to the electronic supplementary material.

Supplementary file1 (DOCX 16 KB)

## Data Availability

No datasets were generated or analysed during the current study.
