# Supplementary material for: Development of Predictive Model of Surgical Case Durations Using Machine Learning Approach
Source: J Med Syst. 2025 Jan 14;49(1):8. doi: 10.1007/s10916-025-02141-y (PMC11732958; doi:10.1007/s10916-025-02141-y)
Supplement: Supplementary file 1 — Supplementary file1 (DOCX 16 KB) [file 10916_2025_2141_MOESM1_ESM.docx]

Table S1. Summary of Surgery and Patient Data Features

| **Feature** | **Type** | **Values/range and statistics** |
| --- | --- | --- |
| Gender | Categorical | Male(45.87%), Female(54.13%) |
| Age | Numeric | Integer (AVG:50.14, STD:24.58) |
| BMI | Numeric | Float(AVG:23.82, STD:14.41) |
| Asa class | Categorical | 2(61.86%), 1(24.77%), 3(12.18%), 4(1.09%), 5(0.06%), 6(0.04%) |
| Condition source value | Categorical | Breast cancer(3.78%), thyroid nodule(1.92%), Ovarian cyst(1.58%), Infiltrating ductal carcinoma of breast(1.56%), Prostate cancer(1.47%)… |
| Surgeon ID | Categorical | 6194(6.67%), 9885(5.13%), 14171(3.34%), 11896(2.94%), 6019(2.25%)… |
| Surgical department | Categorical | Otorhinolaryngology(10.73%), Orthopedics(10.56%), General surgery(9.54%), Obstetrics and gynecology(9.34%), Urology(8.71%), Cardiovascular Thoracic Surgery(6.61%), Neurosurgery(6.53%)… |
| Previous surgery | Categorical | Yes(18.04%) / No(81.96%) |
| Day of the week | Categorical | Monday(20.27%), Tuesday(20.05%), Friday(19.59%), Wednesday(19.39%), Thursday(19.30%), Saturday(0.82%), Sunday(0.56%) |
| Week of the month | Categorical | 2nd (23.61%), 3rd (23.55%), 4th (23.19%), 1st (21.55%), 5th (8.08%) |
| Month | Categorical | January(9.61%), July(9.46%), March(8.92%), April(8.90%), June(8.79%)… |
| Operation code | Categorical | Breast-conserving surgery(3.06%), TUR-B (Transurethral resection-Bladder tumor)(2.49%), Vitrectomy(2.32%), Total hysterectomy(1.94%), TKRA(Total Knee Replacement Arthroplasty)(1.81%)… |
| Anesthesia type | Categorical | general anesthesia(87.78%), spinal anesthesia(8.61%), MAC (monitored anesthesia care)(3.10%)… |
| Emergency status | Categorical | Yes(7%) / No(93%) |
| Operation timing | Categorical | 8A(23.29%), TF1(21.74%), TF2(15.18%), TF3(9.52%), MD(3.17%)… |
| Surgery room | Categorical | B1(5.50%), E1(4.86%), B2(4.68%), E4(4.49%), A3(3.93%), A4(3.49%)… |
| Division | Categorical | Admission(94.09%), Day(5.07%), Outpatient(0.84%) |
| Ward | Categorical | 102(6.86%), NUGW2(6.79%), 72(5.89%), 81(5.84%), 65(4.56%)… |
